# Fluid shear stress induces epithelial-mesenchymal transition (EMT) in Hep-2 cells

**DOI:** 10.18632/oncotarget.8765

**Published:** 2016-04-16

**Authors:** Shuangfeng Liu, Fating Zhou, Yang Shen, Yingying Zhang, Hongmei Yin, Ye Zeng, Jingxia Liu, Zhiping Yan, Xiaoheng Liu

**Affiliations:** ^1^ Institute of Biomedical Engineering, School of Preclinical and Forensic Medicine, Sichuan University, Chengdu 610041, China; ^2^ West China School of Pharmacy, Sichuan University, Chengdu 610041, China; ^3^ School of Medical Laboratory Science, Chengdu Medical College, Chengdu 610500, China

**Keywords:** fluid shear stress (FSS), epithelial-mesenchymal transition (EMT), laryngeal squamous cell carcinomas (LSCC), cell migration

## Abstract

Laryngeal squamous cell carcinoma (LSCC) is one of the most commonly diagnosed malignancies with high occurrence of tumor metastasis, which usually exposes to fluid shear stress (FSS) in lymphatic channel and blood vessel. Epithelial-mesenchymal transition (EMT) is an important mechanism that induces metastasis and invasion of tumors. We hypothesized that FSS induced a progression of EMT in laryngeal squamous carcinoma. Accordingly, the Hep-2 cells were exposed to 1.4 dyn/cm^2^ FSS for different durations. Our results showed that most of cells changed their morphology from polygon to elongated spindle with well-organized F-actin and abundant lamellipodia/filopodia in protrusions. After removing the FSS, cells gradually recovered their flat polygon morphology. FSS induced Hep-2 cells to enhance their migration capacity in a time-dependent manner. In addition, FSS down-regulated E-cadherin, and simultaneously up-regulated N-cadherin, translocated β-catenin into the nucleus. These results confirmed that FSS induced the EMT in Hep-2 cells, and revealed a reversible mesenchymal-epithelial transition (MET) process when FSS was removed. We further examined the time-expressions of signaling cascades, and demonstrated that FSS induces the EMT and enhances cell migration depending on integrin-ILK/PI3K-AKT-Snail signaling events. The current study suggests that FSS, an important biophysical factor in tumor microenvironment, is a potential determinant of cell behavior and function regulation.

## INTRODUCTION

Cancer metastasis is a typical characterization of a malignant tumor, which is the most common cause of cancer-related death. Laryngeal cancer, also known as laryngeal carcinoma, is mostly laryngeal squamous cell carcinomas (LSCC), deriving from the epithelial tissue of the larynx [1]. LSCC can be cured effectively with early diagnosis and treatment. However, clinical evidences showed that LSCC could invade into surrounding tissue and metastasize through the blood and lymph. Recently, the epithelial-mesenchymal transition (EMT) has been recognized as a new source contributing to cancer cell migration and metastasis. Tumor cells progress from non-invasive to malignant phenotypes via a series of critical steps that involve morphological changes referred to as the EMT [2, 3]. EMT which is characterized by the loss of apical basal polarity and tight cell-cell adhesions of epithelial cells and the acquiring of anterior-posterior polarity, migratory and invasive behaviors of mesenchymal cells [4], has been confirmed to play a central role in metastasis of cancers [5, 6].

Loss of E-cadherin (E-Cad) is considered to be a fundamental event in EMT. EMT occurs at the invasive front and produces single migratory cells that lose E-cad expression [7]. Epithelial cells regulate their polarity and cytoskeleton organization, typically express Vimentin filaments, and accumulated β-catenin translocate from cytoplasm to nuclei [8]. Multiple cytokines, chemokines and growth factors including IL-8, FGF and EGF play acritical role as mediators of EMT in cancer cells. Among all the known factors involved in EMT, TGF-β receptors and Wnt/β-catenin induced pathways have taken a center stage [9]. However, the biophysical microenvironment composed by fluid shear stress (FSS) induced EMT is unclear. FSS is exerted on tumor cells exposed to slow interstitial flows in the tumor microenvironment. Fluid mechanics of tumors involve flow along the tumor vasculature, across the vessel wall, through the tumor interstitial space, and drainage by the lymphatic network [10]. LSCC is exposed to low interstitial fluid in the lymphatic channel and blood vessel around tumors, which result in the high occurrence of tumor metastasis. Under physiological conditions in solid tumors, cells may receive interstitial fluid at a velocity of the order of 0.1 to 1 μm/s [11], and shear stress of the order of 0.01 Pa to 0.2 Pa (0.1 to 2 dyn/cm^2^) [12]. The 2 dyn/cm^2^ FSS markedly upregulated matrix metalloproteinase-12 (MMP-12) expression and promotes chondrosarcoma cell invasion [13]. Consequently, we speculate that relatively low FSS can trigger EMT in LSCC, and enhance the migration ability of cancer cells.

FSS activates extracellular elements and adhesion sites linking the cytoplasmic tails of integrins with cytoskeletal proteins [14]. Integrins, which are abundant in the lymph nodes and have been confirmed to be important for lymph node metastasis, play an important role in mediating FSS-induced signals [15]. Both integrins α5β1 and αvβ3 play essential roles in the mechanotransduction of hemodynamic forces into biochemical signals [16, 17]. Integrins are also involved in signal transduction during EMT by stimulating the assembly of intracellular signaling molecules, such as focal adhesion kinase (FAK) or integrin linked kinase (ILK) [18]. Integrin-mediated cell adhesion stimulates the activity of PI3K and its downstream targets of AKT (also known as protein kinase B) and glycogen synthase kinase3β (GSK3β) [19]. Constitutively active AKT in squamous cell carcinoma lines, characterized by down-regulation of E-cad, drive EMT and enhance cell invasiveness by inducing Snail [20]. AKT also contributes to the regulation of Slug-mediated, osteonectin-induced EMT-like changes in melanomas, which emphasizes the crucial role of AKT in controlling Snail family factors and tumor-associated EMT processes [21]. Additionally, FAK plays a central role in the progression of EMT, and the inhibition of integrin/FAK signaling significantly suppresses migration/invasion and growth effects in hepatocellular carcinoma [22].

In the present study, human laryngeal squamous carcinoma Hep-2 cells were exposed to 0.14 Pa (1.4 dyn/cm^2^) FSS for different durations. Cell migration, expression of E-cad and N-cadherin (N-cad), and translocation of β-catenin were characterized to determine the FSS induced EMT in Hep-2 cells. In particular, time-dependent expressions of the intracellular crucial signal molecules in integrin-ILK/PI3K-AKT-Snail and FAs-FAK-Rho GTPases signaling pathways were examined to understand the molecular mechanism involved in FSS-induced development of EMT in Hep-2 cells. Also, Hep-2 cells transfected with ShRNA Snail1 plasmids were further exposed to FSS to determine the role of Snail1 in FSS-induced EMT. This work provides a broad overview of mechanics in tumor microenvironment regulating EMT and establishes associations among FSS, EMT and associated signaling events. Understanding these physical factors can improve therapeutic outcomes in cancers.

## RESULTS

### FSS induced morphology changes of Hep-2 cells

**Figure 1:**
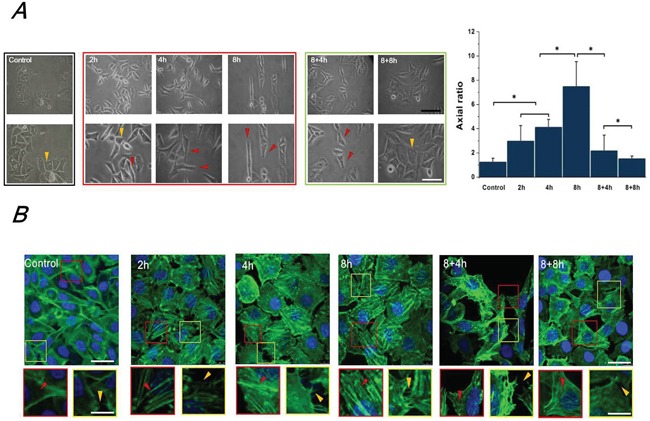
FSS induced the morphology and F-actin changes of Hep2 cells **A.** The morphology of Hep-2 cells exposed to 1.4 dyn/cm^2^ FSS at different durations and observed under 100×(upper) and 200× (lower) magnification using the inverted microscope. The yellow and red marks in Figure 1A indicated the morphology of cloned cells presented polygon and spindle, respectively. The axial ratio of Hep-2 cells was statistically analyzed by Image J. The black and white scale bars in figures are 50 μm and 20 μm, respectively. **B.** Effects of FSS on distribution of F-actin arrays in Hep-2. Confocal immunofluorescence images showed localization of F-actin expose to FSS at 2h, 4h, 8h, in response to removing FSS at 4h and 8h, respectively. (green: F-actin; blue: nucleus, scale bar=20 μm). Designated regions indicated by red/yellow square frames and triangular marks in figures are enlarged to show the detailed fiber structure in the cell body and filopodia/lamellipodia at the edge of cell protrusions (scale bar=5 μm).

The morphologic and cytoskeletal changes of Hep-2 cells induced by FSS were observed by an inverted phase contrast microscope (Figure 1A) and a laser scanning confocal microscope (Figure 1B), respectively. Under a static culture condition (control groups), Hep-2 cells grew cohesively and formed colonies, whereas 1.4 dyn/cm^2^ FSS reduced cell–cell contact and induced protruding filopodia (Figure 1A). With an increased in the duration of exposure to shear stress from 2h to 8h, most cells changed their morphology from polygon (yellow marks in Figure 1A) to elongated fibroblast-like spindles (red marks in Figure 1A). After removal from FSS and static culture for 4h and 8h, most Hep-2 cells gradually recovered their morphology to flat polygon, and formed colonies again (Figure 1A). Image processing and analysis with Image J software, indicates that, the cell aspect ratio (the ratio of major to minor axes) increased with the duration of exposure to FSS, and decreased with FSS removal. Furthermore, the F-actin staining showed that increased duration of FSS stimulation induced more abundant well-organized F-actin in protrusions (lamellipodia and filopodia at the edge of cell protrusions are indicated by red and yellow dashed frames in Figure 1B, respectively). Similarly, following the removal of shear stress (8+4h and 8+8h groups), cells showed fewer and shorter pseudopodia at the leading edge of lamellipodia and displayed disordered actin stress fibers (Figure 1B).

### FSS induced expression and distribution of E-cad and N-cad in Hep-2 cells

**Figure 2:**
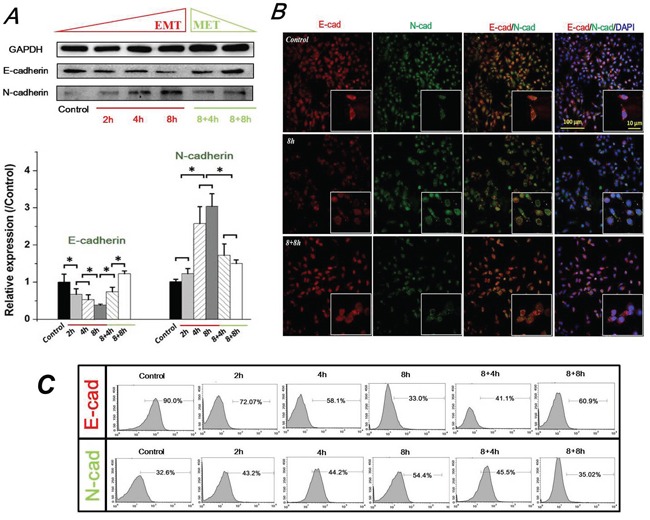
FSS induced expression and distribution of E-cad and N-cad in Hep-2 cells **A.** FSS induced expression of E-cad and N-cad. FSS inducing loss of E-cad led to an EMT process, and a reversible MET occur when FSS was removed. The expression levels of E-cad and N-cad were quantified by image analysis of the Western blot bands. Data are means ± SD from three independent experiments. *, means statistically significant difference with *P*<0.05. **B.** Exposing to FSS induced a changed location of E-cad and N-cad at the boundary of cells. **C.** The positive expression of E-cad and N-cad by flow cytometer at different duration of exposed and removed FSS.

Multiple studies indicated a critical hallmark of the E-cad and N-cad in the epithelial–mesenchymal phenotypic switch of tumor cells during cancer progression. Evidences from these studies confirmed that epithelial cells expressed high levels of E-cad, and E-cad was down-regulated whereas N-cadherin was up-regulated during the process of EMT [2, 7]. Therefore, in this study, the expression levels of E-cad and N-cad were determined by Western blotting (Figure 2A). Our results showed that the epithelial marker E-cad was significantly down-regulated by FSS stimulation at first 2h (*P* <0.05), and continuously decreased at 4h and 8h. However, removing FSS for 4h and 8h induced a recovered up-regulation of E-cad levels in 8+4h and 8+8h groups. On the contrary, exposure to FSS resulted in the mesenchymal marker N-cadherin experiencing a marked up-regulation at 4h, and a significantly increased up-regulation at 8h (*P* <0.05); removing FSS induced the decreased expression of N-cad at 8h (8+8h group). We further investigated the distribution of E-cad and N-cad by immunofluorescence. As shown in Figure 2B, Hep-2 cells in controls (without exposure to FSS) showed a high positive expression of E-cad. The enlarged images indicate that red fluorescence (marked E-cad) showed higher intensity than green fluorescence (marked N-cad) at the edge of cells. Exposing to FSS for 8h resulted in a decreased expression of E-cad and occupied location of N-cad at the boundary of cells (Figure 2B). These immunofluorescence results were consistent with the results of Western blotting (Figure 2A). The flow cytometry (FCM) results also confirmed the regularity of E-cad and N-cad expression induced by FSS. The positive expression of E-cad decreased from 90% in the control group to 33.0% in the 8h group, and increased to 60.9% in the 8+8h group, whereas the positive expression of N-cadherin increased from 32.6% in the control group to 54.4% in the 8h group, and dropped to 35.02% in the 8+8h group, similar to control. These results demonstrated that exposure to FSS triggered an EMT process in Hep-2 cells, whereas removing FSS led to a reversal mesenchymal-epithelial transition (MET) event in a time-dependent way.

### FSS induced translocation of β-catenin in Hep-2 cells

**Figure 3:**
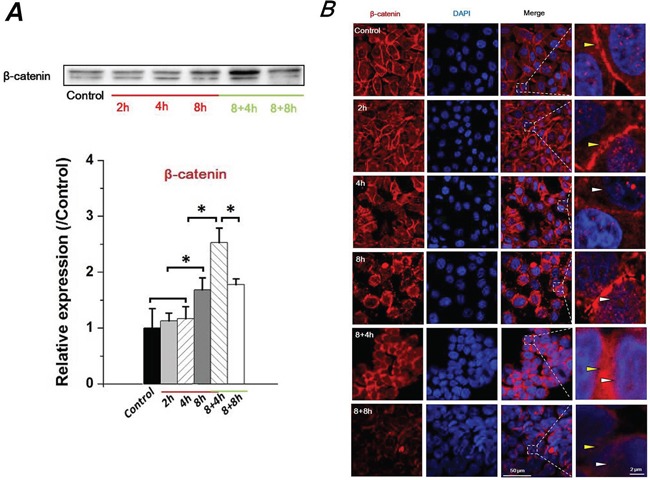
FSS induced expression level change and translocation of β-catenin in Hep-2 cells **A.** FSS induced a changed expression of β-catenin in time-dependent manner. β-catenin level was quantified by image analysis of Western blot bands. *, means statistically significant difference with *P*<0.05. **B.** The presence or absence of FSS induced a translocation of β-catenin. FSS markedly induced an accumulation of β-catenin in nucleus (white marks), but translocated to cytomembrane (yellow marks) when FSS was removed.

Recent studies have demonstrated a key role of the E-cad/β-catenin signaling axis for EMT involving epithelial cells [23]. As an important component of adherens junction, β-catenin binds the cytoplasmic tail of E-cad, and links to the actin cytoskeleton in conjunction with α-catenin [24]. Loss of E-cad expression results in the translocation of accumulated β-catenin from the cytoplasm to the nucleus, and nuclear β-catenin induces a gene expression pattern favoring tumor invasion and facilitating EMT [25]. Thus, the expression and distribution of β-catenin regulated by FSS were examined (Figure 3). The expression level of β-catenin significantly increased at 8h with 1.4 dyn/cm^2^ FSS stimulation. Interestingly, it was continuously up-regulated in 8+4h groups and down-regulated in 8+8h groups (Figure 3A). Additionally, FSS markedly induced accumulation of β-catenin in nuclei (white marks); then β-catenin was translocated from the nucleus to membranes (yellow marks) after removal of FSS (Figure 3B). These findings confirmed that FSS was a determinant of EMT and MET mediation in Hep-2 cells.

### FSS triggered the EMT process of Hep-2 cells and enhance their migration ability

**Figure 4:**
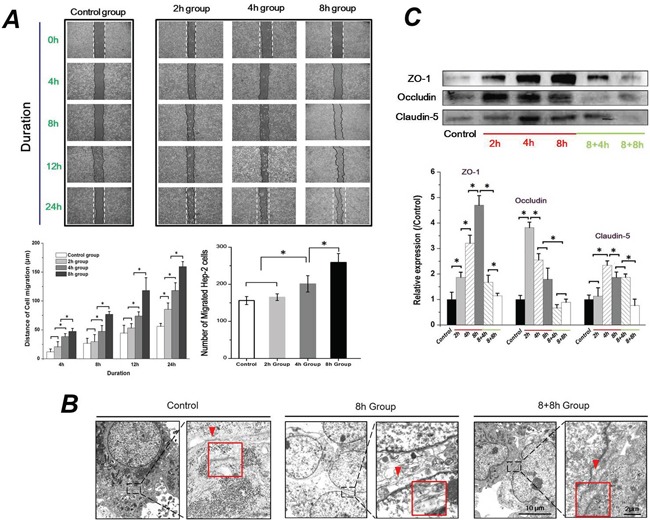
Fluid shear stress enhanced cell migration ability and changed cell-cell junctions **A.** Exposed to FSS enhanced Hep-2 cell migration ability in a time-dependent manner. The 8h group (Hep-2 cells were exposed to FSS for 8h) showed the largest migrated distances and maximum migrated cell number at 24h, compared with control, 2h and 4h group. **B.** The TEM images showed that FSS decreased cell-cell junctions. The red marks and enlarged frames showed the junctions and gaps between two cells. The scale bars in TEM images of each group are 10μm and 2μm with gradual enhanced magnification (5000×and 20,000×). **C.** The effect of FSS on Occludin, Claudin-5 and ZO-1 expression. The expression levels were quantified and statistically analyzed by image analysis of Western blot bands. *, means statistically significant difference with *P*<0.05.

To investigate the migration ability of Hep-2 cells during the process of EMT, the Hep-2 cells were exposed to 1.4 dyn/cm^2^ FSS for 2h, 4h and 8h (named as 2h group, 4h group and 8h group, respectively). Then, slides with 100% confluence of cells were taken out from the flow chamber, and a 500-μm uniform scratch was performed with a plastic cell scrapper. As seen in Figure 4A, the migration ability of Hep-2 cells was gradually enhanced with increasing exposure duration to FSS. Data analyses revealed that the cell migration distance of 4h and 8h groups was significantly longer than that of the 2h and control groups at initial 4h to final 24h (*P* <0.05). There was no significant difference between cell migration distance of 2h and control groups at 12h (*P* <0.05), although 2h groups showed a longer cell migration distance than control groups at 24 h. Also, statistical analysis indicated that 8h groups showed the highest number of migrated cells across the baseline (initial injured wound, indicating by dashed line in figure) compared to 2h, 4h and control groups (Figure 4A). These results suggested that Hep-2 cells with mesenchymal transition enhanced their migrated ability, depending on duration of exposure to FSS.

Down-regulated genes and proteins of tight junction (TJ) components (ZO-1, Claudin-5 and Occludin) is considered as one of the early events in EMT. The disassembly of TJ results in disruption of the cell polarity complex and initiation of cytoskeleton reorganization [26]. We thus observed the morphological changes of TJ at the closely associated areas of two cells using TEM, and examined the protein levels of ZO-1, Claudin-5 and Occludin. The open gap at the TJ could be clearly found with exposure to FSS for 8h (red arrows indicated in Figure 4B); closed space appeared when FSS was removed for 8h (8+8h group), which was similar to control group (Figure 4B). The protein expression level of Occludin, Claudin-5 and ZO-1 were further examined by a Western blotting assay (Figure 4C). Interestingly, statistical results revealed that Occludin expression showed a sharp up-regulation at the initial 2h of FSS stimulation, and subsequently decreased at 4h; the expression of Claudin-5 increased at 4h and subsequently decreased at 8h. FSS induced a gradual increase in the level of ZO-1 in Hep-2 cells from 2h to 8h. Removing FSS resulted in a sharp down-regulation of ZO-1 expression (Figure 4C).

### FSS triggered the EMT in Hep-2 cells, depending on the integrins signaling event

**Figure 5:**
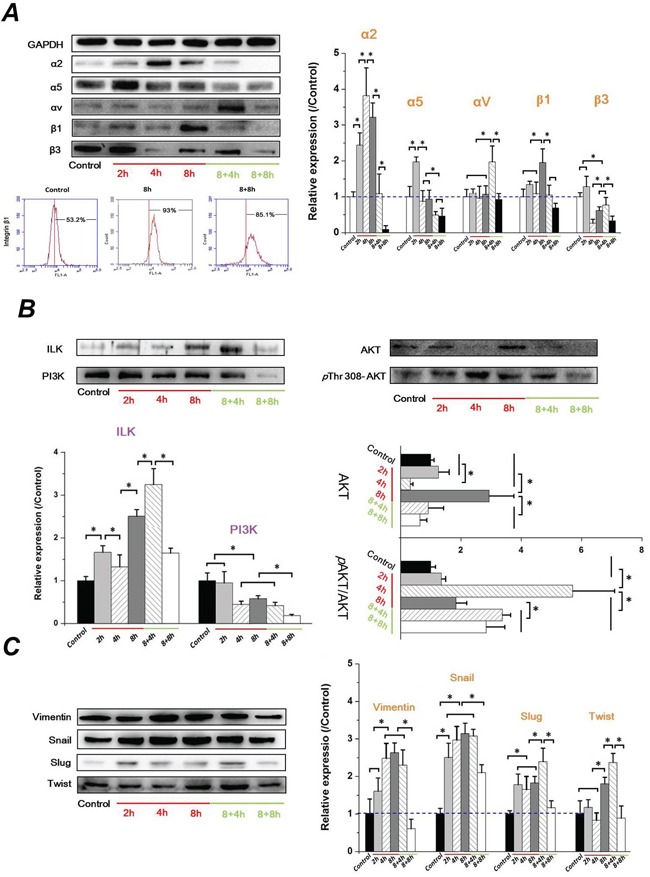
FSS-induced EMT in Hep-2 cells depended on integrins signaling events FSS regulated the expression change of **A.** integrins α and β subunits, **B.** ILK/PI3K/AKT signaling and, **C.** mesenchymal markers (Vimentin, Snail, Slug, and Twist). The expression levels were quantified and statistically analyzed by image analysis of Western blot bands. *, means statistically significant difference with *P*<0.05.

It is well-known that the transmembrane receptors, α subunits of integrin serve as mechanosensors, which respond to fluid shear stress in vascular endothelial cells and then integrate and transduce the signaling via intracellular β subunits [27]. Increased evidence indicates an important role of integrin signaling in regulating EMT and controlling tumor cell migration and invasion [28, 29]. ILK interacts directly with cytoplasmic domains of the β1 or β3 integrin subunits [30], and contributes to stabilize the expression of E-cad expression and the translocation of β-catenin to the nucleus [31]. To determine if the FSS inducing the EMT in Hep-2 cells depends on the integrins signaling event, the expressions of integrin α (α2, α5, αV) and β subunits (β1 and β3) were investigated. As shown in Figure 5A, there were significant differences of integrin α and β subunits among different durations of FSS stimulation. Both α2 and α5 integrin subunits increased during the first 2h and decreased subsequently. The only difference was that α2 subunits reached a peak at 4h compared with α2 subunits at 2h. Interestingly, there was no difference in the expression of αV integrin from 0h (control) to 8h. However, the level of αV sharply increased in the 8+4h group. Additionally, the β1 subunit showed a stronger expression at 8h, and subsequently decreased to a similar expression level of the control group. The FCM results also confirmed the regularity of integrin β1 expression induced by FSS. The expression of β1 increased from 53.2% in the control group to 93.0% in the 8h group, and subsequently decreased to 85.1% in 8+8h group.

Differing from other integrin subunits, the level of β3 showed no marked changes during the initial 2h exposed to FSS, but it was significantly down-regulated at 4h (*P* <0.05). Subsequently, it showed an up-regulation when FSS was removed for 4h (8+4h groups); eventually it decreased to a much lower expression eventually (8+8h) (Figure 5A). These differences suggested that different roles of integrin subunits participated in FSS regulating EMT in Hep-2 cells. Integrating the signals from TGF-β and β integrin, the expression of ILK increased with duration of exposure to FSS, and was down-regulated with increasing time following removal of FSS, which was consistent with results of N-cad and β-catenin. In contrast, the expression of PI3K decreased with increased duration of exposure to FSS (Figure 5B). Both of PI3K and ILK have been suggested to phosphorylate and activate downstream AKT at Thr 308 and Ser 473 in a PI3K-dependent or -independent manner [32]. The phosphorylated AKT is important for nuclear localization and protein stabilization of Snail and Slug [33, 34]. As shown in Figure 5B, the expression of AKT in Hep-2 cells exposed to FSS showed a sharp change from 2h to 8h. There was no significant difference of AKT between 2h and control group (*P*>0.05). The AKT level decreased at 4h but rapidly recovered at 8h. Subsequently, it gradually decreased with removal of FSS. Additionally, the ratio of phosphorylation level of AKT (*p* AKT) at Thr 308 in total AKT (*p* AKT/AKT) showed the peak at 4h (Figure 5B). Figure 5C showed that both of the expression of Snail and Slug was up-regulated markedly at 2h, and down-regulated when the FSS was removed for 8h. Similar to Snail and Slug, the mesenchymal marker Vimentin and Twist significantly increased at 4h and 8h, respectively, and also decreased in 8+8h group. These results indicated that FSS induced EMT in Hep-2 cells in a time-dependent way.

### FSS triggered the EMT in Hep-2 cells depend on the FAK signaling

**Figure 6:**
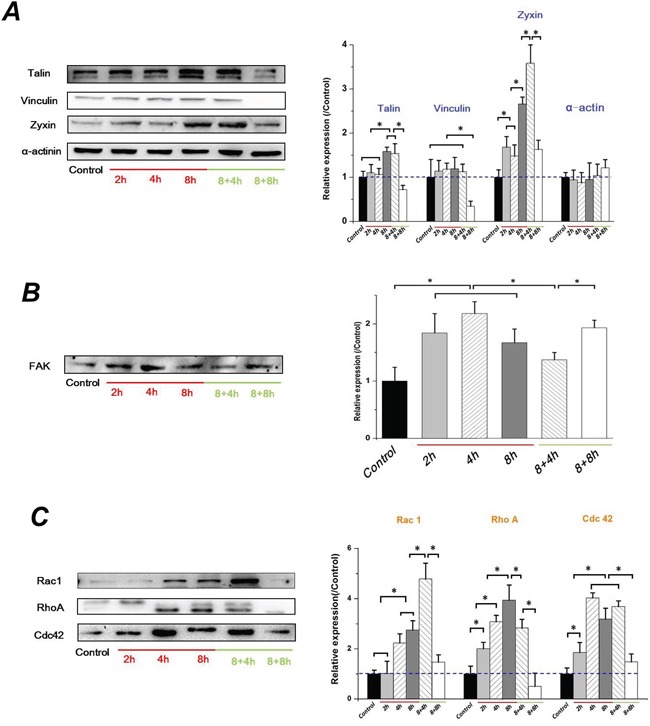
FSS-induced EMT in Hep-2 cells depended on FAK signaling cascade **A.** The F-actin changing by FSS in Hep-2 cells depended on the assembly of cytoskeletal proteins, Talin, Vinculin, Zyxin and α-actinin. **B.** FSS regulated the expression change of FAK. **C.** The Hep-2 cells with EMT enhance their migration ability depended on increased expression level of Rho-family GTPases (RhoA, Rac1 and Cdc42). The expression levels were quantified and statistically analyzed by image analysis of Western blot bands. *, means statistically significant difference with *P*<0.05.

FAK is involved in integrin-mediated cell signaling and overexpression in various epithelial cancers [35]. The activation of FAK triggers multiple intracellular signaling pathways to regulate various cellular functions, and plays a key role in TGFβ-mediated EMT [36]. The assembly of cytoskeletal proteins such as Talin, Vinculin, and Paxillin, form actin-rich structures of FA, bind to FAK and contribute to support a stable cell-matrix adhesion. As seen in Figure 6A, α-actinin level in Hep-2 cells showed no significant difference with increased exposure and removal of FSS. However, the Talin and Zyxin expression in Hep-2 cells showed a similar tendency in that they were significantly increased at 8h and maintained for 4h without FSS, but were down-regulated at 8h in 8+8h group. Similarly, the expression of Vinculin showed a sharp decrease at 8h in 8+8h group, but no significant differences with FSS for 8h and without FSS for 4h (Figure 6A). The expression of FAK increased at first 4h and decreased at subsequent 8h, and continuously down-regulated when FSS was removed at initial 4h. Interesting, the FAK recovered to a higher level in 8+8h groups, equal to the level at 2h (Figure 6B). Rho-family GTPases (RhoA, Rac1 and Cdc42) result in direct local actin assembly to form stress fiber, lamellipodia or filopodia, respectively. They act together to regulate cell motility through their effects on the cytoskeleton, membrane trafficking and cell adhesion [37]. The expression of RhoA was enhanced with increased duration of exposure to FSS, and was reduced with FSS removal. The expression of Rac1 and Cdc42, are implicated in cell polarization during cell migration, and showed a similar tendency in that they were increased from 2h and maintained a relative higher level even if FSS was removed for 4h, but were sharply down-regulated at 8h in 8+8h group (Figure 6C). The FSS up-regulated expression levels of RhoA, Rac1 and Cdc42 in a time-dependent manner, which was consistent with previous results of F-actin changes (Figure 1B) and cell migration (Figure 4A), suggesting that FSS induced the EMT and enhanced the migration ability of the Hep-2 cells via Rho-family GTPases.

### Sh-Snail1 inhibits the FSS-induced EMT in Hep-2 cells

**Figure 7:**
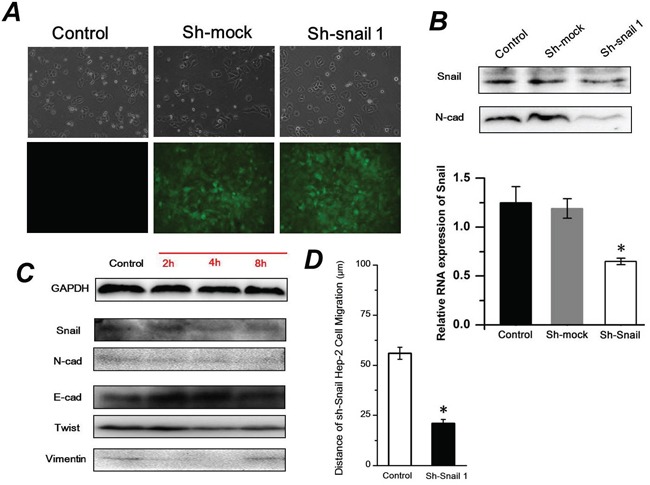
Sh-Snail1 inhibits the FSS-induced EMT in Hep-2 cells **A.** The differences of cell morphology among the Hep-2 cells transfected with shRNA-mock or shRNA snail1 plasmids and control using inverted phase contrast microscope. The green fluorescence indicated that plasmids had been successfully transfected into Hep-2 cells using fluorescence microscope. **B.** Snail1 and N-cadherin expression levels following inhibition of Snail1 in Hep-2 cells analyzed by western blotting and RT-PCR. **C.** FSS induced expression of Snail, E-cad, N-cad, Twist and Vimentin in Sh-Snail Hep-2 cells. **D.** Wound scratch assay revealed that the cell migration distance in shRNA Snail1 was significantly shorter than that of control group after 24h.

It is known that snail transcription factors promote the repression of the adhesion molecule E-cadherin to activate EMT. To elucidate the role of snail in FSS-induced EMT, the ShRNA Snail1 plasmid was applied to knockdown the expression of Snail1 in Hep-2 cells. There was no distinct difference of cell morphology among the Hep-2 cells transfected with shRNA-mock or shRNA snail1 plasmids and control group. The green fluorescence indicated that plasmids had been successfully transfected into Hep-2 cells (Figure 7A). The RT-PCR and Western blot results confirmed the efficacy of Snail-1 shRNA. The expression levels of N-cad in the Hep-2 cells transfected with the Snail-1-shRNA vector were significantly weaker than those in the Hep-2 cells with control group or control shRNA (shRNA-Mock) (Figure 7B). Subsequently, the Hep-2 cells transfected with the Snail-1-shRNA were exposed to 1.4 dyn/cm^2^ FSS for 2, 4 and 8h. As shown in Figure 7C, knockdown of Snail resulted in persistently higher levels of E-cad and lower N-cad expression with increased duration of FSS. Additionally, the shRNA Snail1 led to a remarkable downregulation of mesenchymal marker Vimentin. Both Vimentin and Twist in shRNA-Snail Hep-2 cells induced by FSS showed in a time-independent way. The wound scratch assay revealed that the cell migration distance in shRNA Snail was significantly shorter than that of control group after 24h (Figure 7D). These results suggested that knockdown of Snail impeded FSS induction of EMT in Hep-2 cells.

## DISCUSSION

### Tumor mechanical microenvironment triggers EMT

The tumor microenvironment is a cellular environment including tumor cells, immune cells, fibroblasts, lymphocytes, signaling molecules and growth factors. The solid stress exerted by the interactions among these cellular components, and the microvascular and interstitial fluid shear stress exerted by blood and lymphatic flow, constitute together a complicated tumor mechanical microenvironment [38]. When exerted directly on cancer cells, shear stress can increase cells’ invasive and metastatic potential. Wang P and Guan PP et al indicated that shear stress with 2 dyn/cm^2^ increased the release of cyclic AMP and interleukin-1β via the induction of MMPs (MMP-1, MMP-7 and MMP-12) in PI3-K and ERK1/2-dependent manner, which significantly promoted motility and invasion of chondrosarcoma cells [39, 40].

EMT has been well-documented in multiple cancer cell models and is believed to be one of the earliest events in tumor progression. Although not proven, EMT has been recognized as a potential mechanism to explain cancer progression and metastasis. In tumor microenvironments, a variety of cytokines and growth factors, such as TGF-β [41], have been previously confirmed to trigger EMT. Interestingly, Grabias et al revealed that shear stress (2 and 4 dyn/cm^2^) increases type I collagen deposition and reduces cell motility and transmigration of proximal tubular epithelial cells (PTECs), suggesting that shear stress did not induce an EMT process in proximal tubular epithelial cells [42]. On the contrary, Rizvi et al's results demonstrated that a flow transcriptionally regulated decrease in E-cad expression and a simultaneous increase in vimentin in epithelial ovarian cancer cells, indicating increased metastatic potential [43]. At the laryngeal squamous carcinoma, LSCC is exposed to FSS in lymphatic channel and blood vessels, which are indeed an area with a high occurence of tumor metastases. To understand whether FSS would accelerate the metastasis of laryngeal squamous carcinoma, the effect of FSS on EMT progression and consequent cell migration were investigated in this study.

### Mesenchymal Hep-2 cells enhanced migration ability

The process of EMT in cells results in the loss of epithelial properties, and acquisition of mesenchymal properties. The epithelial derived LSCC lose their cell polarity and cell-cell adhesion, and gain migratory and invasive properties to become mesenchymal-like cells. Historically, epithelial and mesenchymal cells have been identified on the basis of their unique visual appearance and the morphology of the multicellular structures. Mesenchymal cells have a more extended and elongated shape relative to epithelial cells, and they possess front-to-back leading edge polarity [44]. Turning an epithelial cell into a mesenchymal cell requires alterations in many *in vitro* functional markers, including cell morphology, cellular architecture, adhesion, and migration capacity [45]. In our study, we found that the 1.4 dyn/cm^2^ FSS induced an obvious morphologic change of Hep-2 cells from polygon to spindle; the shape of cells would be returned to polygons 8h following removal of FSS (Figure 1A). During EMT, polymerization of the actin cytoskeleton is clearly observed. Shankar et al [46] indicated that Cytochalasin D (Cyt D) reduced cell size and F-actin levels and induced an up-regulation of E-cad at both protein and mRNA level. We found in addition that the actin cytoskeleton was rearranged with FSS stimulation. With increased exposure to FSS, more lamellipodia and filopodia could be found at the edge of cell protrusions, and well-organized F-actin was abundantly accumulated in cell body (Figure 1B), suggesting that cells could achieve a migratory and invasive phenotype for crossing tissue barriers and thereby reaching blood and lymphatic vessels. It is known that FSS can induce a cell morphology change and F-actin rearrangement, independent of EMT. It is difficult to determine whether the changed morphology and enhanced migration was induced by EMT process or by FSS. Accordingly, we further examined the expression of mesenchymal and epithelial markers to support a conclusion that FSS induced an EMT process in Hep-2 cells.

EMT is accompanied by decreased Claudin and Occludin expression. Cell–cell junctions between neighboring cells and cell-substrate adhesions hold epithelial cells tightly together, while mesenchymal cells showed weaker adhesions than their epithelial counterparts, allowing for increased migratory capacity [45]. The TJ, which is located at the top side of the gap of adjacent cells, is formed by a variety of proteins and represents the first barrier that cancer cells must overcome in order to metastasize. The occurrence of EMT is always accompanied by the disruption of TJ. To further explore the effect of FSS on cell–cell junctions between Hep-2 cells, we observed morphological changes of TJ at closely associated areas between cells with TEM (Figure 4B), and quantitatively detected the expression of TJ components in Hep-2 cells (Figure 4C). The identified components of TJ include several families such as Claudins, Occludin, junctional adhesion molecules (JAMs), and cytosolic protein Zonula occludens (ZO). An important TJ component ZO-1, functions as a bridge between TJ and the actin cytoskeleton, which is part of a multi-protein complex binding F-actin and the integral TJ proteins Occludin and Claudin [47]. Studies showed that the decreased expression of ZO-1 correlated with the EMT process and increased invasiveness in cancers [48]. Interesting, the expression of ZO-1 was up-regulated with increased exposure to FSS in our study. Additionally, down-regulation of occludin has also been shown to lead to elevated levels of progression and metastatic potential in cancers [49], and it also might be the reason for activating of EMT and a consequent down-regulation of adhesion associated proteins [50]. Kokudo et al [51] indicated that TGFβ2 induced differentiation of embryonic stem cell derived endothelial cells with a decrease in the expression of claudin 5. In this study, we found that Occludin and Claudin-5 showed a sharply increased expression exposed to FSS for 2h and 4h, respectively, but they both decreased at 8h. Cucullo et al [52] showed that 6.2 dynes/cm^2^ shear stress increased the RNA levels of a variety of tight and adherens junction components in microvascular endothelial cells, including ZO-1 and Claudin 5, which could promote the formation of a tight and highly selective of blood-brain barrier. Therefore, FSS could transiently up-regulate the level of TJ components. It is speculated that exposure to FSS for longer durations induced the EMT in Hep-2 cells, and simultaneously resulted in decreased expression of Occludin and Claudin. However, our results showed an increase in expression of ZO-1 with FSS stimulation for 8h. This pattern needs to be further investigated.

We further find the evidences that FSS induced the molecular markers of EMT. An important characteristic of EMT is the loss of E-cad expression resulting in destabilization of the cell-cell contacts and detachment of cells from their surroundings. In addition, β-catenin binds directly to the intracellular domain of E-cad to α-catenin. β-catenin connects the adherens junction complex with the actin cytoskeleton [53], and could transduce the Wnt signaling from the cell surface to the nucleus. When Frizzled family members accepted the Wnt signal at the cell surface, β-catenin accumulates in the nuclei and then activates transcription of Wnt responsive genes [54]. Our results showed that FSS down-regulated E-cad, and simultaneously up-regulated N-cadherin (Figure 2A), which was further verified with results of immunofluorescence (Figure 2B) and flow cytometry (Figure 2C). The expression and translocation of β-catenin (Figure 3A & 3B) and the expression of mesenchymal markers, Vimentin and Twist (Figure 5C) confirmed the conclusion that FSS induces the EMT in Hep-2 cells.

### Cellular signals associated with FSS inducing EMT

Transcription program switching in EMT is mediated by TGF-β, EGF, FGF, Wnt/β-catenin, Notch, Hedgehog, and integrin signaling pathways. These pathway signals induce transcription factors that activate the expression of EMT-associated genes through intracellular kinase cascades [55]. Integrins are increasingly recognized for their structure in mechanosensor and mechanotransduction [56], and they play an important role in the EMT process. It was demonstrated that integrin α2β1 interacting with collagen type I could lead to a loss of E-cad in pancreatic cancer cells, and that activation of α2β1 integrin by collagen type I could induce N-cadherin expression [57]. We speculated mechanisms by which integrins are involved in the progression of FSS inducing EMT, and demonstrate that different roles of integrin subunits participated in EMT in Hep-2 cells. Our results indicated that the integrin α2 and β1 were up-regulated during FSS that induced EMT, which is consistent with previous results showing that the activated integrin α2β1 could lead to the up-regulation of N-cadherin and down-regulated of E-cad [57, 58], suggesting the occurrence of EMT. ILK is a β1-integrin cytoplasmic domain-interacting protein acting as a scaffold protein, aiding in the formation of protein complexes, connecting integrins to the actin cytoskeleton and signaling pathways, and functioning as the effector of PI3K si gnaling [59]; it is also a signaling protein involved in the regulation of cell survival, proliferation, and migration [19]. A recent study indicated that ILK/β-parvin/cofilin signaling pathways contributes to the initial formation of filopodium-like protrusions of carcinoma cells in EMT programming [60]. Our results also demonstrated that abundant well-organized F-actin in protrusions and more lamellipodia and filopodia at the edge of cell protrusions could be found with increased duration of exposure to FSS (Figure 1B), accompanied with the nuclear translocation of β-catenin (Figure 3B) and up-regulation of ILK (Figure 5B). On the contrary, the down-regulation of ILK resulted in a decrease of lamellipodia and filopodia when FSS was removed. This result confirmed a switch between EMT and MET in Hep-2 cells with or without FSS treatment. PI3K/AKT is emerging as another axis of EMT, which could activate Snail and Slug, and regulate the expression and distribution of E-cad [21, 61]. Consequently, the opposite tendency of ILK and PI3K expression possibly led to a sharp change of AKT level from 2h to 8h (Figure 5B). AKT induces activation of GSK-3β, which in turn suppresses phosphorylation of Snail to induce EMT. Participation of Akt/GSK-3*β*/Snail pathway in the acquisition of EMT phenotype has been reported previously [62]. In this study, FSS up-regulated both Snail and Slug, that may suppress the expression of E-cad and promote EMT. The loss of E-cad expression is often associated with the increased nuclear localization and activity of β-catenin, whereas Snail and Slug could be further up-regulated by the Wnt-β-catenin pathway, and further promoted the EMT [34, 63].

During EMT, cancer cells change their adhesive characteristics, and gradually gain migratory and invasive properties that involve a dynamic reorganization of the actin cytoskeleton. An integrin-mediated actin cytoskeleton is accomplished by a multiprotein complex consisting of the adaptor proteins talin, vinculin and ILK. The interaction and phosphorylation status of FAK and Src play a central role in assembly and turnover of the integrin complex [18]. By mediating cytoskeletal organization and focal adhesion turnover, Vimentin was proved to contribute to mechanics of cancer cells in EMT [64]. Sperry et al [65] indicated that Zyxin functioned in morphogenetic rearrangements and controlled migration in EMT by mediating actin-membrane linkages at cell-cell junctions. Our results demonstrated that FSS regulated the expression of Vimentin (Figure 5C), Talin and Zyxin (Figure 6A) in an identical manner; i.e., they significantly increased at 8h, and maintained at a higher level even if FSS was removed for 4h (in 8+4h group), but they were sharply down-regulated at 8h (in the 8+8h group).

The wound healing assay confirmed that FSS enhanced the migration capacity of the Hep-2 cells (Figure 4A). The Rho-GTPases family, Rac, Rho and Cdc42, are well-studied regulators of cell motility. Rac induces the assembly of focal complexes and actin polymerization during the formation of lamellipodia. Rho induces the formation of stress fibers, whereas Cdc42 induces actin polymerization for the formation of filopodia. For most cell types, the acquisition of a more motile phenotype during EMT has been attributed to the increased expression and activation of Rho GTPases [66]. The Rac1, RhoA and Cdc42 were up-regulated by FSS (Figure 6C), which further proved that the FSS can induce the migration ability of Hep-2 cells by actin cytoskeleton reorganization, suggesting that FSS induces EMT in Hep-2 cells.

## MATERIALS AND METHODS

### Cell culture and FSS loading

The human laryngeal squamous carcinoma Hep-2cell line was purchased from Shanghai institutes for biological sciences (SIBS) in the Chinese Academy of Sciences. The Hep-2 cells were cultured in 1640 medium (Invitrogen, California, USA) supplemented with 10% fetal calf serum at 37°C in 5% CO_2_.

The cells were dissociated enzymatically with 0.25% trypsin, and then resuspended in 10% fetal serum-containing media. Cell density at 1.0×10^5^ cells/mL was determined with a haemocytometer and added to a polystyrene tissue culture plates cultured with the sterilized glassslides (25.4 mm × 76.2 mm × 0.2 mm). Until 90% confluence on the glassslides, the Hep-2 cells were exposed to stable uniform0.14 Pa (1.4 dyn/cm^2^) FSS for 2, 4, or 8h (defined as 2h group, 4h group and 8h group, respectively, as shown in Table 1) using a parallel flow chamber. After 8h, FSS was cancelled, and two groups were taken out from flow chamber and cultured at static conditions for 4h and 8h, defined as 8+4h and 8+8h groups (Table 1). The static cultured Hep-2 cells on the glass slide without FSS stimulation were set as the control group.

**Table 1:** Sample identification codes and preparation conditions

Identification Codes	Fluid shear stress (dyn/cm^2^)	Stimulated duration of Shear stress	Static culture after cancelled shear stress
**Control** group	-	-	-
**2h** group	1.4	2h	-
**4h** group	1.4	4h	-
**8h** group	1.4	8h	-
**8+4h** group	1.4	8h	4h
**8+8h** group	1.4	8h	8h

### Wound-healing assay

Cell migration was measured by a wound-healing assay. The Hep-2 cells were cultured on the glass slides until monolayer confluence. The glass slides were placed into a flow chamber and exposed to 1.4 dyn/cm^2^ FSS for 2h, 4h and 8h. Then a uniform scratch (about 500-μm width) was performed in the cell monolayer using a plastic cell scrapper. The glass slides with scratch injury were re-cultured in an incubator containing 5% CO_2_ at 37°C. Each group (2h, 4h and 8h) were photographed consecutively at 0, 4, 8, 12h and 24h using an inverted microscope (CK2, Olympus, Japan). The distance of cell migration was determined by calculating the difference between the end length and the original length edge using the Image J software.

### Western blotting assay

Hep-2 cells cultured on the glass-slides were washed twice by PBS on ice, and then were lysed by RIPA buffer with 1% Phenylmethanesulfonyl fluoride (PMSF) and protease inhibitor for 30 min. After centrifugation at 12,000 rpm for 5 min, the supernatant was collected and quantified by BCA (bicinchoninic acid) Protein Assay Kit (Beyotime Institute of Biotechnology, China). Equal amounts of lysate were separated by SDS-PAGE and transferred to PVDF membranes. Membranes were blocked in 1% BSA at room temperature for less than 2h, and then incubated with the diluted primary antibodies in TBST buffer at 4°C for overnight. Detailed information of primary antibodies, blocking and incubation conditions is shown in Table 2.

**Table 2:** List of antibodies used in this study

Category	Primary antibody name	Isotype	Blocking Conditions 1-hr room temperature	Primary Ab incubation	Manufacturer
E-cad/N-Cad	E-cadherin (H-108): sc-7870	rabbit pAb mouse mAb	5% nonfat dry milk in PBS	Overnight at 4°C, 1:200 dilution	Santa Cruz, Inc
	N-cadherin (H-4):sc-271386	mouse mAb	5% nonfat dry milk in PBS	Overnight at 4°C, 1:100 dilution	
Tight Junction components	Occludin (E-5): sc-133256	mouse mAb	5% nonfat dry milk in PBS	Overnight at 4°C, 1:200 dilution	Santa Cruz, Inc
	Claudin-5 (H-52):sc-28670	rabbit pAb	5% nonfat dry milk in PBS	Overnight at 4°C, 1:200 dilution	
	ZO-1 (H-300):sc-10804	rabbit pAb	5% nonfat dry milk in PBS	Overnight at 4°C, 1:100 dilution	
Integrins	α2integrins (C-9):sc-74466	mouse mAb	5% nonfat dry milk in PBS	Overnight at 4°C, 1:200 dilution	Santa Cruz, Inc
	α5 integrins (A-11):sc-166665	mouse mAb	5% nonfat dry milk in PBS	Overnight at 4°C, 1:200 dilution	
	αV (H-2):sc-376156	mouse mAb	5% nonfat dry milk in PBS	Overnight at 4°C, 1:100 dilution	
	β1 (A-4):sc-374429	mouse mAb	5% nonfat dry milk in PBS	Overnight at 4°C, 1:200 dilution	
	β3 (B-7):sc-46655	mouse mAb	5% nonfat dry milk in PBS	Overnight at 4°C, 1:200 dilution	
ILK/PI3K/(*p*) AKT	ILK (65.1):sc-20019	mouse mAb	5% nonfat dry milk in PBS	Overnight at 4°C, 1:200 dilution	Santa Cruz, Inc
	PI3K C2β (16L9):sc-100407	mouse mAb	5% nonfat dry milk in PBS	Overnight at 4°C, 1:100 dilution	
	AKT1 (B-1):sc-5298	mouse mAb	5% nonfat dry milk in PBS	Overnight at 4°C, 1:200 dilution	
	*p* AKT1 (Thr 308):sc-135650	rabbit pAb	3% BSA in PBS	Overnight at 4°C, 1:200 dilution	
	Vimentin (V9): sc-6260	mouse mAb	5% nonfat dry milk in PBS	Overnight at 4°C, 1:200 dilution	Santa Cruz, Inc
	Snail (H-130): sc-28199	rabbit pAb	5% nonfat dry milk in PBS	Overnight at 4°C, 1:100 dilution	
	Slug (A-7): sc-166476	mouse mAb	5% nonfat dry milk in PBS	Overnight at 4°C, 1:200 dilution	
	Twist (Twist2C1a): sc-81417	mouse mAb	5% nonfat dry milk in PBS	Overnight at 4°C, 1:100 dilution	
β-catenin	β-catenin (E-5): sc-7963	mouse mAb	5% nonfat dry milk in PBS	Overnight at 4°C, 1:100 dilution	Santa Cruz, Inc
FA components	Talin (8D4):sc-59881	mouse mAb	5% nonfat dry milk in PBS	Overnight at 4°C, 1:200 dilution	Santa Cruz, Inc
	Vinculin (G-11):sc-55465	mouse mAb	5% nonfat dry milk in PBS	Overnight at 4°C, 1:200 dilution	
	α-actinin (H-2):sc-17829	mouse mAb	5% nonfat dry milk in PBS	Overnight at 4°C, 1:200 dilution	
	Zyxin (H-200):sc-15338	rabbit pAb	5% nonfat dry milk in PBS	Overnight at 4°C, 1:100 dilution	
FAK	FAK (A-17):sc-557	rabbit pAb	5% nonfat dry milk in PBS	Overnight at 4°C, 1:200 dilution	Santa Cruz, Inc
Rho GTPases	Rac1 (C-11):sc-95	rabbit pAb	5% nonfat dry milk in PBS	Overnight at 4°C, 1:200 dilution	Santa Cruz, Inc
	RhoA (26C4):sc-418	mouse mAb	5% nonfat dry milk in PBS	Overnight at 4°C, 1:200 dilution	
	Cdc42 ab64533	rabbit mAb	5% nonfat dry milk in PBS	Overnight at 4°C, 1:100 dilution	Abcam®, Inc

After washing with TBST buffer three times, membranes were incubated with the appropriate peroxidase-conjugated secondary antibody for 2h. Enhanced chemiluminescence (ECL) was used for detecting the target proteins, and blots were imaged by Molecular Image® ChemiDoc™ XRS+ with Image Lab™ Software (Bio-Rad Laboratories, Inc, USA). The tests were performed three times and quantification was analyzed by Image J 1.44p software. The intrinsic controls (GAPDH) were used to guarantee that protein was loaded equally among all groups.

### Analysis of cell-cell junction by transmission electron microscope (TEM)

The Hep-2 cells were exposed to FSS for 8h. Then cells were statically cultured for another 8h without FSS. The cell samples (control, 8h and 8+8h groups) were collected and centrifuged at 1,200 rpm for 10 min, fixed with 0.5% glutaraldehyde and stored at 4°C for 10 min. The samples were centrifuged again at 12,000 rpm for 10 min and the supernatant was discarded. Then 3% glutaraldehyde was slowly added. All samples were fixed by 1% OsO_4_, dehydrated by increasing concentrations of acetone, embedded by epoxy resin (Epon812), cut into slices and doubled dyed using uranyl acetate and lead citrate. The micro-structure of cell-cell junctions were observed by TEM (H-600IV, Japan).

### Immunofluorescence staining and flow cytometry

The expression and the distribution of E-cad, N-cad, β-catenin were further presented by immunofluorescence (IF) staining. Each group of Hep-2 cells was washed twice by PBS for 5 min, fixed with 4% paraformaldehyde at room temperature for 8 min and permeabilized with 0.3% Triton X-100 for 10 min. Then, samples were washed three times with PBS and subsequently blocked with 1% BSA for 15 min. The cells were incubated with rabbit or mouse polyclonalprimary antibodies (E-cad, N-cad, β-catenin) at 4°C overnight. After washing the slide three times for 5 min, the FITC-conjugated goat anti-mouse antibody or PE-conjugated goat anti-rabbit antibody were incubated with the cells for 90 min. DAPI (4′ 6′-diamidino-2-phenylindole) was used to stain nuclei for 30 min at 37°C. Cells were sealed by 10% glycerol, kept in a dark place, and detected by laser scanning confocal microscope (Leica TCS SP5, Germany).

Each group of Hep-2 cells was digested with 0.25% trypsin and then washed with PBS. 100 μL of 10^6^ cell suspension in PBS was placed in a 15 ml centrifuge tube at RT for 1h with primary anti-E-cad and anti-N-cad. Following PBS washes, bound antibody was detected using FITC-conjugated goat anti-mouse antibody or PE-conjugated goat anti-rabbit antibody, and analyzed by flow cytometry (FACSAria II, Becton Dickinson, USA).

### ShRNASnail1 and Transfection

The plasmids of ShRNA snail1 containing reporter gene EGFP and the puromycin resistance cassette were designed and synthesized from Genecopoeia Inc (Rockville, MD, USA). The effective plasmid of ShRNA snail1 was filtered, which is listed in Table 3. Hep-2 cells were cultured in a 6 well plate until they reached 80% confluence; they were then transfected by ShRNA snail1 sequence for 24h with Attractene Transfection Reagent Kit according to the manufacturer's protocol (QIAGEN, Germany), and then selected by 0.4 μg/mL puromycin until stable clone formation. The transfection efficiency was determined by fluorescence intensity. The stable clones were dissociated and cultured in 1640 medium (Invitrogen, California, USA) containing 10% fetal serum and 0.2 μg/mL puromycin with 90% confluence for examinations.

**Table 3:** The detailed information of Snail shRNA Plasmids

Clone Name	Symbol	Location	Length	Target Sequence
HSH017573-8-LVRH1GP (OS396160)	SNAI1	1465	19	GAGTAATGGCTGTCACTTG

### Statistical analysis

All experiments were repeated three times and presented as mean ± standard deviation. Data obtained from different treatment groups were statistically compared. To reveal differences among the groups, one-way ANOVA followed by Tukey's test was used. Differences were considered significant at *P* <0.05.
